# Seasonal Changes in the Endosymbiotic Consortia of Aphids from the Genus *Cinara*

**DOI:** 10.1264/jsme2.ME15118

**Published:** 2016-06-07

**Authors:** Vanesa Martínez-Díaz, Amparo Latorre, Rosario Gil

**Affiliations:** 1 Institut Cavanilles de Biodiversitat i Biologia Evolutiva (ICBiBE), Universitat de ValènciaC/Catedrático José Beltrán 2, 46980 Paterna (Valencia)Spain; 2 Fundación para el Fomento de la Investigación Sanitaria y Biomédica de la Comunidad Valenciana (FISABIO)Avenida Cataluña 21, 46020 ValenciaSpain

**Keywords:** *Buchnera aphidicola*, *Serratia symbiotica*, *Cinara tujafilina*, *Cinara cedri*, Temperature

## Abstract

*Buchnera aphidicola* is the primary endosymbiont of aphids with which it maintains an obligate mutualistic symbiotic relationship. Insects also maintain facultative symbiotic relationships with secondary symbionts, and *Serratia symbiotica* is the most common in aphids. The presence of both symbionts in aphids of the subfamily *Lachninae* has been widely studied by our group. We examined two closely related aphids, *Cinara tujafilina* and *C. cedri* in the present study. Even though both *B. aphidicola* strains have similar genome sizes and gene contents, the genomes of the two *S. symbiotica* strains were markedly different. The SCc strain has the smallest genome known for this species, while SCt possesses a larger genome in an intermediate stage between the facultative *S. symbiotica* of *Acyrthosiphon pisum* (SAp) and the co-obligate *S. symbiotica* SCc.

Aphids are vulnerable to high temperatures. Previous studies indicated that *S. symbiotica* SAp confers resistance to heat-shock stress. In order to clarify whether *S. symbiotica* strains from genus *Cinara* also play a role in heat stress protection, we performed a quantitative determination of the consortium *Buchnera/Serratia* from two geographically close populations, each of which belonged to the *Cinara* species examined, over two years in natural environments. We found no variation in the consortium from our *C. cedri* population, but a positive correlation between both endosymbiont densities and average daily temperatures in the *C. tujafilina* population. Even though *S. symbiotica* SCt may retain some protective role against heat stress, this does not appear to be due to the release of protective metabolites by cell lysis.

Mutualistic symbiosis between intracellular bacteria and insects is widespread in nature and appears to be one of the keys to the evolutionary success of this group of organisms (34). In most known cases, bacteria supply essential nutrients that are absent or scarce in unbalanced host diets (*e.g.*, plant sap, vertebrate blood, and cereals). Based on their essentiality for host survival and fitness, two classes of insect endosymbionts have been identified, *i.e.*, obligate or primary (P) and facultative or secondary (S).

Obligate mutualists are confined within specialized cells called bacteriocytes, and are transmitted strictly vertically from the mother to offspring. As expected from this lifestyle, they maintain highly constrained relationships with their hosts (51). In aphids, the P-endosymbiont *Buchnera aphidicola* has established long-term obligate mutualistic symbiosis (31). It provides essential amino acids in order to complement the phloem diet of its host, which is poor in nitrogen compounds (8). This mutualistic relationship has been supported by genomic (7, 22, 28, 41, 46, 47, 50), transcriptomic (16), and proteomic (42) analyses of several *B. aphidicola* strains belonging to aphids from different subfamilies.

Facultative endosymbionts are not essential for host survival, and beneficial, harmful, or neutral effects have been described (12, 39, 40). They have established a more recent association with the host than P-endosymbionts. Although they are also vertically transmitted, horizontal transfer events between hosts have also been reported (18, 39, 43). They may live in different locations inside the host, such as sheath cells, the hemolymph, secondary bacteriocytes, or surrounding primary bacteriocytes. Three main S-symbionts have been described in aphids, *i.e.*, *Hamiltonella defensa*, *Regiella insecticola*, and *Serratia symbiotica* (32), with the latter being the most widely distributed among different aphid lineages (22, 43, 53). Most experimental studies have been performed on the pea aphid *Acyrthosiphon pisum* (subfamily *Aphidinae*), in which the three aforementioned S-symbionts have been found (44), and different beneficial effects have been proposed. All three common facultative symbionts of *A. pisum* are known to be involved in protection against parasitoid wasps (37, 45), while *R. insecticola* provides resistance to the fungal pathogen *Pandora neoaphidis* (45). *R. insecticola* and *H. defensa* may also contribute to host plant specialization (14, 18, 49), while the main benefit provided by *S. symbiotica* is protection against heat stress (6, 30, 44).

The genome sequencing of *S. symbiotica* SAp (from *A. pisum*, subfamily *Aphidinae*) corroborated the facultative status of this symbiont (4). *S. symbiotica* is prominent in members of the subfamily *Lachninae* (3, 22) and has been characterized in detail in two aphid species from the genus *Cinara*, the cedar aphid *C. cedri* (Mimeur, 1936) and thuja aphid *C. tujafilina* (del Guercio, 1909) (23, 29). Although both *Cinara* species are clustered as sister clades, distantly related with the Aphidinae, phylogenetic analyses on *S. symbiotica* showed that SAp and SCt (from *C. tujafilina*) belong to the same clade (clade A), while *S. symbiotica* from other Lachninae (including SCc from *C. cedri*) belong to a different one (clade B; 22, 29). In *C. cedri*, the genome sequencing of *S. symbiotica* SCc revealed that it has taken over some of the roles that *B. aphidicola* plays in other aphids. Thus, it is necessary to complement *B. aphidicola* BCc for the synthesis of tryptophan (15) and it is responsible for the synthesis of vitamins and cofactors (23). Therefore, both endosymbionts are considered to be co-primary. On the other hand, the genome sequencing of *S. symbiotica* SCt and its comparison with *S. symbiotica* SCc and the facultative *S. symbiotica* SAp revealed that it is in an intermediate stage between them (29). Moreover, while *S. symbiotica* SCt is rod-shaped, may be found extracellularly, and possesses a large genome (2.5 Mb), resembling the facultative strain *S. symbiotica* SAp (4), *S. symbiotica* SCc is always confined inside bacteriocytes, has a pleomorphic shape similar to *B. aphidicola*, and has the smallest genome known for this species, with a gene content similar to that of other P-endosymbionts (23). Nevertheless, the pathway for the biosynthesis of riboflavin was shown to be lost in the ancestor of *B. aphidicola* from the *Cinara* species, causing the fixation of *S. symbiotica* as an obligate endosymbiont (29). Differences between both *S. symbiotica* strains are marked, considering *B. aphidicola* strains BCc and BCt have similar genome sizes and gene numbers (24).

One important factor in determining the geographical distribution of a species is the range and variability of temperatures tolerated by it. Since they are poikilothermic, insects are very sensitive to extreme temperatures. Temperature not only has direct effects on insect hosts, but also on their symbionts, as demonstrated in field and laboratory experiments (9, 13, 33, 44, 48). In the case of aphids, in addition to their difficulty tolerating high temperatures (6, 36), the thermal sensitivity of their obligate endosymbiont *B. aphidicola* further constrains the tolerance of the host (51). Several hypotheses have been proposed to explain the high susceptibility of the endosymbionts to heat stress, and are mainly related to the special genomic features of these microorganisms (51). They typically have a very high AT content, leading to more thermosensitive DNA molecules (52), but may also affect the stability of structural RNAs. The accumulation of non-synonymous substitutions in endosymbiont genomes due to their accelerated mutation rates may cause higher thermal susceptibility in the encoded proteins. Additionally, their reduced genomes have lost most genes encoding cell envelope proteins, potentially resulting in fragile cells.

Studies conducted on *A. pisum* demonstrated that heat stress reduced the number of bacteriocytes, causing important decreases in *B. aphidicola* densities (19, 30, 36), which has marked consequences on host fitness. However, the concomitant presence of *S. symbiotica* (and, to a lesser extent, *H. defensa*) may improve aphid fitness under heat stress (2, 6, 17, 30, 44). Previous studies suggested that the facultative symbiont enhances the preservation of bacteriocytes and, thus, the survival of *B. aphidicola* at high temperatures (30, 44). Consequently, *S. symbiotica* infections are highly prevalent in pea aphid populations after periods of summer heat or in hot desert sites (30, 51).

If the vulnerability of obligate endosymbionts to heat stress limits the tolerance of the host to temperature changes (51), the transition of *S. symbiotica* from a facultative to an obligate lifestyle may have reduced its ability to protect the aphid host from heat stress. The different level of mutualistic integration of the two *S. symbiotica* strains from *Cinara* aphids offers a good opportunity to test this hypothesis. In order to investigate whether *S. symbiotica* SCc and SCt play a role in heat stress protection, and if their presence affects *B. aphidicola* density, we performed a quantitative determination on the consortium *Buchnera/Serratia* from a single population of each *Cinara* species under study over two years in natural conditions in the Mediterranean area of Valencia (Spain).

## Materials and Methods

### Insect rearing and sampling

*C. cedri* specimens were collected from a permanent population that was established from individuals collected in 2011 (23) and maintained since then on two cedar trees of the species *Cedrus atlantica glauca* in the facilities of the ICBiBE at the University of Valencia (Paterna, Valencia, Spain. 39° 30′ 58″ N, 0° 25′ 20″ W; 24). *C. tujafilina* aphids were collected on April 14, 2012 from thuja bushes of the species *Platycladus orientalis* from Paiporta (Valencia, Spain. 39° 25′ 52″ N, 0° 24′ 59″ W), and were used to infect two thuja bushes grown in 5-L pots (20×20×19 cm^3^), which were initially maintained in Paiporta and transferred to the facilities of the ICBiBE on June 17, 2013. Trees and bushes were searched for adult aphids on the plant stem and leaves twice a week in 2012, and weekly in 2013. When adult aphids were scarce, we also included L4 instar insects in the sampling. Daily hourly temperatures in the closest meteorological station (8414A Valencia/Aeropuerto, 39° 29′ 06″ N, 0° 28′ 29″ W) were obtained from the Spanish National Meteorology Agency (AEMET; http://www.aemet.es). Since samples were always taken early in the morning, the temperatures considered for each sample were those recorded the day before. The temperature regime was characterized using the following daily parameters: maximum temperature (T_max_), minimum temperature (T_min_), average temperature (T_ave_), temperature oscillation (T_osc_), and the standard deviation of temperatures (SD_T_).

### Total DNA extraction and quantification

Total insect DNA (_T_DNA) was extracted immediately after sampling using the method of Latorre *et al.* (26) with slight modifications. Approximately 6.5 mg of insects was gently homogenized in 160 μL buffer I (10 mM Tris-HCl [pH 7.8]; 60 mM NaCl; 5% sucrose; 10 mM EDTA) at 4°C. After the addition of 200 μL of buffer II (300 mM Tris-HCl [pH 8.0]; 1.25% SDS; 5% sucrose; 10 mM EDTA), the mixture was incubated at 65°C for 30 min, neutralized with 60 μL 3 M potassium acetate (pH 5.0), and kept at −20°C for 20 min. _T_DNA was concentrated by precipitation using standard protocols, resuspended in ultrapure water, and stored at −20°C until its use. The concentration and quality of _T_DNA were measured using a PicoGreen dsDNA Quantification Assay (Invitrogen [Thermo Fisher Scientific], Waltham, MA, USA).

### Quantitative real-time PCR (qPCR)

The abundance of *B. aphidicola* and *S. symbiotica* in the two *Cinara* species under study was determined by qPCR on a LightCycler 2.0 with 20-μL capillaries using the LightCycler FastStart DNA Master^PLUS^ SYBR Green I Kit (Roche Diagnostics) according to the manufacturer’s instructions.

Multiple alignments were performed in ClustalW (25; http://www.clustal.org) to detect adequate sequence regions for the design of specific primer pairs to the single-copy genes *gro*EL from *B. aphidicola* and *atp*D from *S. symbiotica* (Table 1). The gene *atpD* was selected for the quantification of *S. symbiotica* because it is not present in any of the *B. aphidicola* strains under study (24, 41). The sequences of the genes under study were retrieved from GenBank (Acc. No. for the complete genomes of *B. aphidicola* BCc, NC_008513.1; *B. aphidicola* BCt, CP001817.1 and *S. symbiotica* SCc, CP002295.1. Acc. No. of the scaffolds of the *S. symbiotica* SCt genome containing the genes *atp*D, FR904236.1, and *gro*EL, FR904230.1). Primer pairs were designed with IDT’s Oligo Analyzer 3.1 (http://eu.idtdna.com/analyzer/Applications/OligoAnalyzer). By taking advantage of the sequence similarity between the *gro*EL orthologues from *B. aphidicola* BCc and BCt, a single primer pair was designed for both bacterial strains. The specificity of each primer set was checked by BLAST (1, http://blast.ncbi.nlm.nih.gov/Blast.cgi) and empirically by gel electrophoresis, as recommended by the MIQE guidelines (5).

We used recombinant plasmid DNA as quantification calibrators. Each primer set was used for conventional PCR amplification with the KAPATaq DNA Polymerase Kit (Kapa Biosystems, Woburn, MA, USA) using insect _T_DNA as a template. The thermal cycling protocol was as follows: initial denaturation at 95°C for 2 min, followed by 30 cycles at 95°C for 30 s, at 60°C for 30 s, and at 72°C for 1 min; a final extension at 72°C for 2 min. Amplicons were purified with the High Pure PCR Product Purification Kit (Roche), and cloned into a pGEM-T Easy Vector (Promega, Madison, WI, USA). The recombinant plasmids pGEM-groELBCc, pGEM-groELBCt, pGEM-atpDSCc, and pGEM-atpDSCt were purified using the High Pure Plasmid Isolation Kit (Roche). Plasmid concentrations were measured using a PicoGreen dsDNA Quantification Assay (Invitrogen). Calibration curves were obtained according to Lee *et al.* (27). The regression lines for the standard curves had a mean squared error <0.1.

**Fig. 1:**
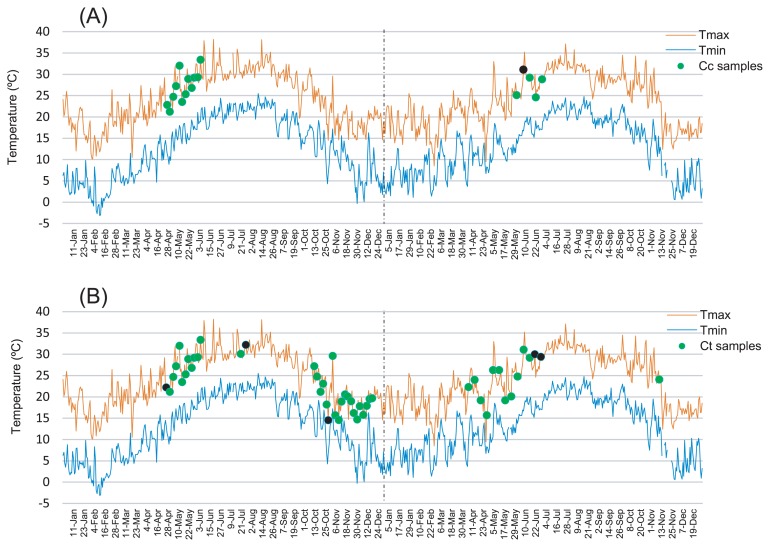
Maximum (upper line) and minimum (lower line) daily temperatures recorded in Valencia airport (Manises, Valencia) meteorological stations by AEMET in 2012 and 2013. The days in which the aphid samples were taken are indicated with dots. (A) *C. cedri*. (B) *C. tujafilina*. Black dots indicate that aphids were collected, but the samples were not used for further analyses.

qPCR experiments were performed in a total volume of 10 μL, using 0.5 μM of each primer, and 1 μL of _T_DNA as the template. Each experiment was performed simultaneously for both target genes, in two biological replicates, and in three technical replicates containing 2.0, 1.0, and 0.5 ng μL^−1^ of _T_DNA, respectively. Each technical replicate was analyzed twice. Following MIQE guidelines (5), qPCR reactions containing water or the corresponding recombinant plasmid instead of _T_DNA were also performed for each primer pair as negative and positive controls, respectively. qPCR reaction conditions were as follows: one cycle of 95°C for 10 min, followed by 35 cycles of 95°C for 5 s, 60°C for 6 s, and 72°C for 7 s. At the end of each run, the fidelity of the amplification was checked through a melting-curve analysis for each amplicon, with a temperature gradient of 0.1°C s^−1^ from 70 to 95°C. The technical replicates for each dilution had a mean squared error ≤ 0.2. The amplification results were examined by electrophoresis on 1.4% agarose gels. The efficiency of each biological replicate was calculated after each qPCR run, and only replicates with efficiencies equal to or greater than 1.7 were taken into consideration. Therefore, one *C. cedri* sample (June 10, 2013) and five *C. tujafilina* samples (April 29, July 29, October 31 in 2012; June 24 and July 1 in 2013) were excluded from subsequent analyses (black dots in Fig. 1).

### Statistical Analysis

In order to rule out that a putative effect of temperature on the variable examined was spurious and actually due to aphid biomass, we initially examined the relationship between aphid weight (mg) and each of the temperature parameters under study. We found no correlation between the aphid biomass and any of the parameters used as parameters of the temperature regimes (Pearson’s correlation coefficients, r: −0.133 to 0.234, *p*>0.1). Similarly, in order to test the putative effect of the aphid biomass on the log-transformed relative proportion of the symbionts (*gro*EL copy number/*atpD* copy number), we used an ANCOVA model with host species as a fixed factor with two levels (*C. tujafilina* population, Ct; *C. cedr*i population, Cc) and the aphid biomass as a covariate. In the model, we assumed that the regression coefficient relating the dependent variable and the covariate varied with the factor level (*i.e.*, interactions: host x aphid biomass).

The relationship between the log-transformed relative proportion of the symbionts, on one hand, and the host species and temperature regimes, on the other, was analyzed as follows. Since high communality is expected for the temperature parameters, we performed pairwise correlation analyses for each parameter under consideration in order to select a sound number of independent variables and avoid noise introduced by redundancy. Using *p*≤0.01, we identified two groups of variables, one composed by T_max_, T_min_, and T_ave_ (r: 0.735 to 0.949), and the other by T_osc_ and SD_T_ (r>0.96). The maximum r was 0.37 for parameters in different groups (not significant). These results keep for the two sets of measurements (*C. cedri* and *C. tujafilina* populations). We selected T_ave_ and T_osc_ as representatives of the first and second groups, respectively, because they have a straightforward interpretation. T_ave_ and T_osc_ were then used as covariates for an ANCOVA, similar to that described above, except for the covariates included in the model. In this case, we tested for the interactions host x T_ave_ and host x T_osc_.

In order to examine changes causing variations in the relative proportion of the symbionts, the response of both symbiont abundances to temperature was investigated using a linear regression analysis. Four regression analyses (two hosts x two symbionts) were computed on log abundances using T_ave_ as the independent variable.

All analyses were performed using SPSS (IBM Corp. Released 2013. IBM SPSS Statistics for Windows, Version 22.0. Armonk, NY: IBM Corp).

## Results and Discussion

Aphids from the genus *Cinara* infest coniferous trees. In cold climate regions, most species have holocyclic lifecycles. They overwinter as eggs that hatch in the spring into asexual females, which produce a succession of several generations of viviparous parthenogenetic females during the favorable seasons. In the fall, they produce a single annual generation of sexual individuals, which mate and lay overwintering eggs. However, in warm climate regions such as the Mediterranean area, some species are anholocyclic, and the adult parthenogenetic females survive in suboptimal temperature conditions (too cold or too warm) by migrating onto the plant’s trunk or to the roots, the temperatures at which are milder (35). *C. cedri* lives in compact colonies on the twigs, branches, and trunks of cedars. When the temperature is too cold or too warm, adults hide under the tree bark or on the roots, and reappear at the beginning of spring. *C. tujafilina* mainly infests the twigs of thuja trees. In studies performed in Poland, adult aphids were found in large numbers in spring, but also at the beginning of autumn when temperatures range between 15 and 25°C, with an optimum at 25°C. The nymphs migrate to the roots when adverse temperatures are maintained for more than 72 h, while the adults remain on the trunk until death (10, 11). In the present study, even though we searched for aphids on the plant stem and leaves twice a week in 2012, and weekly in 2013, we were only able to detect *C. cedri* between May and June in 2012 and in June, 2013 (Fig. 1A). As for *C. tujafilina*, we were able to collect aphid samples for a longer period of time, between May and July and between October and December in 2012, and between April and July in 2013 (Fig. 1B). An additional sample was collected in November that year. Differences in the presence of aphids between 2012 and 2013 may have been due to the unusual weather conditions in 2013 in the Mediterranean area, according to data collected by AEMET, with a cool spring, which delayed the onset of *C. cedri* colonies on the tree branches, and warm autumn, which delayed the migration of *C. tujafilina* to the twigs after summer.

**Fig. 2:**
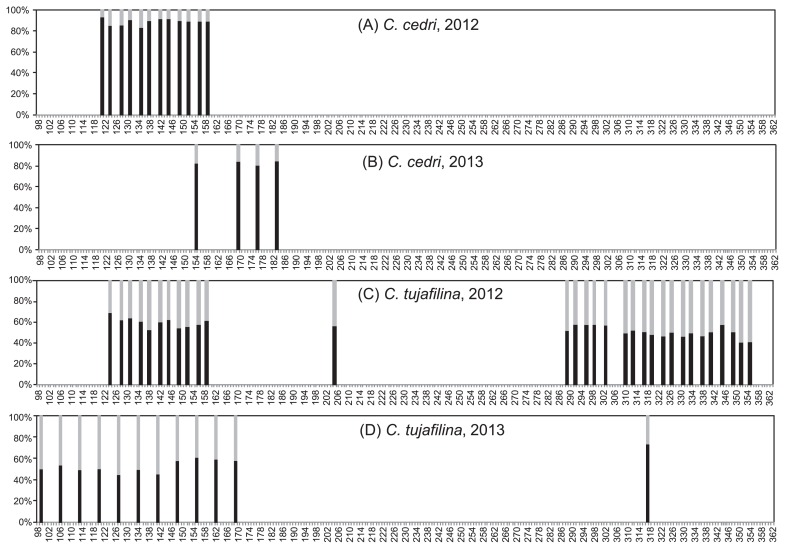
Relative quantification of *B. aphidicola* and *S. symbiotica* from *C. cedri* and *C. tujafilina* in 2012 and 2013. The x-axis indicates the time (d, Tables 2 and 3), the y-axis indicates the percentage of each endosymbiont (*B. aphidicola* in black, *S. symbiotica* in grey).

**Fig. 3:**
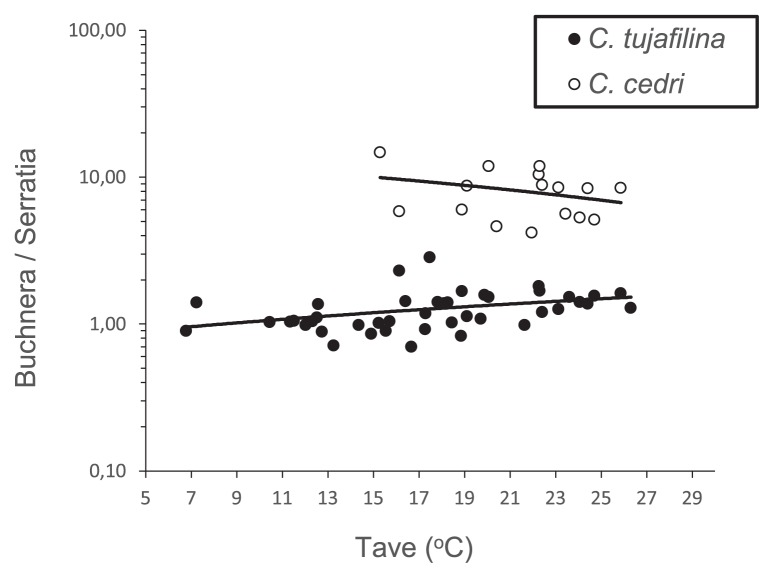
Relationship between the ratio ‘*gro*EL copy number/*atp*D copy number’ and daily average temperature for *C. tujafilina* and *C. cedri* populations over two years under study.

We determined endosymbiont densities in our samples using qPCR with the single copy genes *gro*EL and *atp*D as indicators of the amount of each endosymbiont in the consortia in both aphid species under study. The results obtained are shown in Tables 2 and 3 (for *C. cedri* and *C. tujafilina*, respectively). We observed differences in the relative abundance of *B. aphidicola* and *S. symbiotica* depending on the host (Fig. 2 and 3). In *C. cedri*, *B. aphidicola* BCc was always present at a higher proportion, and only slight variations among samples were observed (between 80.7 and 93.6%), with an average of 89.33% in 2012 and 82.99% in 2013 (Fig. 2A and 2B). In contrast, relative amounts were highly variable in *C. tujafilina* (Fig. 2C and 2D; Table 3). *B. aphidicola* BCt oscillated between 69.80% and 41.08% in 2012, and between 73.93% and 45.30% in 2013.

Since the individual insect size differed in the different samples, we first tested whether there was a relationship between insect weight and the relative abundance of *B. aphidicola* and *S. symbiotica* in both aphid species. We found that the two aphid species populations analyzed differed significantly in their average values for the log-ratio ‘*Buchnera*/*Serratia*’ (ANCOVA, *p*<0.001) after controlling for aphid weight, whereas the log-ratio did not correlate with aphid weight in the two species under study (ANCOVA, *p*=0.372 for Ct, 0.057 for Cc).

We then investigated the influence of temperature on the relative amount of both endosymbionts, using the daily average temperature and temperature oscillations as independent variables. The results obtained revealed that the two aphid species populations analyzed differed significantly in their average values for the log-ratio ‘*Buchnera*/*Serratia*’ (ANCOVA, *p*<0.001) after controlling for T_ave_ and T_osc_. However, while the changes observed in the log-ratio did not correlate with T_osc_ in the two species (ANCOVA, *p*>0.175), the relationship of this log-ratio with T_ave_ was dependent on the host species (ANCOVA, *p*=0.024; Fig. 3). In *C. cedri*, slight changes in this log-ratio did not correlate with T_ave_ (ANCOVA, *p*=0.190), whereas these changes positively correlated in *C. tujafilina* (ANCOVA, *p*=0.016).

**Fig. 4:**
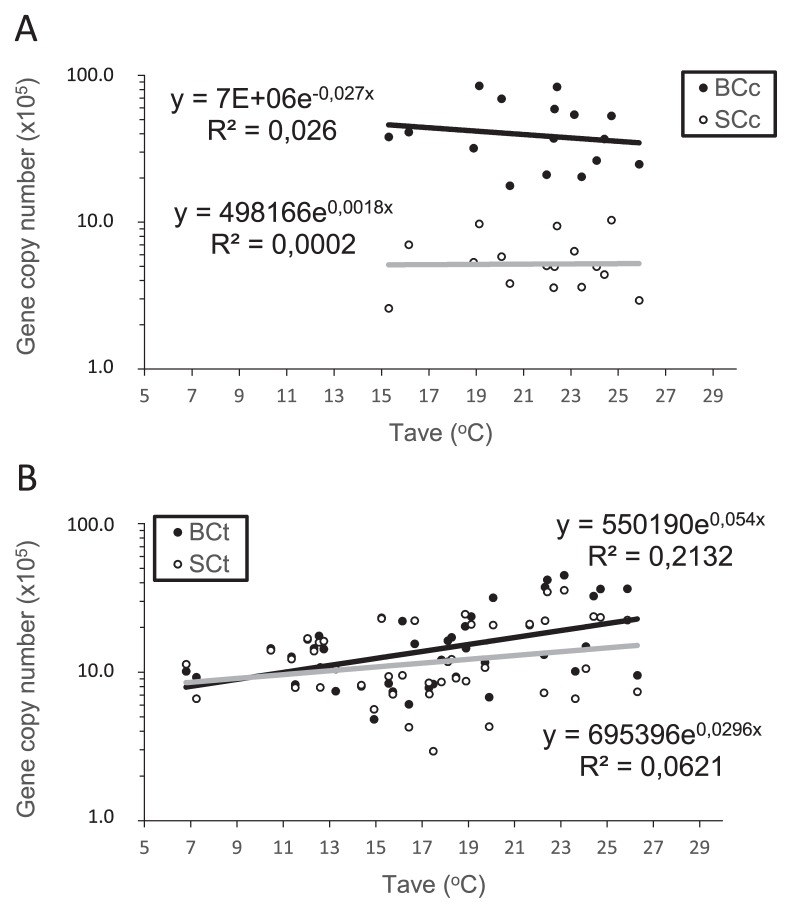
Linear regression between the gene copy number (*gro*EL and *atp*D) and daily average temperature for two aphid species populations under study over two years. (A) *C. cedri*. (B) *C. tujafilina*.

In the case of *C. cedri*, a linear regression analysis of the response of endosymbiont abundance (measured as copy numbers of *gro*EL from *B. aphidicola* and *atp*D from *S. symbiotica*) to T_ave_ indicates that the cell densities of both endosymbionts did not correlate with daily average temperatures (Fig. 4A; *p*=0.551 for *B. aphidicola* BCc; *p*=0.960 for *S. symbiotica* SCc). These results suggest that *S. symbiotica* SCc does not have a protective role in *B. aphidicola* BCc, as expected, because this *S. symbiotica* strain presents a highly reduced gene content, similar to other obligatory endosymbionts (23). This may compromise the ability of *C. cedri* to adapt to high temperatures, which may, in turn, result in the disappearance of this species from the Mediterranean area as a consequence of climate change.

On the other hand, the behavior of *C. tujafilina* indicates that it still has some potential to cope with high temperatures. Based on knowledge on *A. pisum*, if *S. symbiotica* SCt retains some protective role against heat stress, we speculate that it will tolerate the consequences of climate change in the Mediterranean area better. Burke *et al.* (2) analyzed pea aphid populations, with and without protective S-symbionts, exposed to heat stress. They corroborated that when *A. pisum* was infected with *S. symbiotica*, *B. aphidicola* cell density was not altered in response to heat exposure; however, many *S. symbiotica* cells simultaneously lysed. Therefore, the density of *S. symbiotica* was reduced to one half after a heat shock treatment at 39°C. However, in the *C. tujafilina* population analyzed in the present study, a statistical analysis of the absolute copy numbers of *gro*EL and *atp*D revealed that although both endosymbiotic bacteria showed a trend line with a positive slope (0.0540 for *B. aphidicola* BCt; 0.0296 for *S. symbiotica* SCt; Fig. 4B), a significant positive correlation was only observed between the average temperature recorded and the amount of *B. aphidicola* BCt (*p*=0.002). This result indicates that the increase observed in the *Buchnera*/*Serratia* ratio in *C. tujafilina* (Fig. 3) was due to an increase in *B. aphidicola* BCt abundance, and not to a decrease in *S. symbiotica* SCt cell density. Nevertheless, the increase found in *B. aphidicola* BCt cell density with higher daily average temperatures may be in accordance with the hypothesis of a protective role for *S. symbiotica* SCt. It is important to note that, as in previous studies (2, 38), we used single copy genes as indicators of the amount of each endosymbiont in the consortia. Although the ploidy of *S. symbiotica* has not yet been investigated, *B. aphidicola* from *A. pisum* has the ability to possess more than 100 genome copies cell^−1^ (20), and the genome copy number varies in response to the developmental stage and morph of the host (21). Even though we used aphids from the same developmental stage, the possibility of an increase in the *B. aphidicola* BCt gene copy number due to genome amplification in response to heat stress cannot be ruled out. In any case, an increase in the number of genomes may also be linked to an increase in gene function.

Based on metabolomic analyses, it was hypothesized that the lysis of *S. symbiotica* after heat exposure may deliver protective metabolites that enhance *B. aphidicola* survival (2). The authors suggested that molecules such as N-acetyl- D-mannosamine, mannose-6-phosphate, and β-alanine are involved in this function. Since no decrease occurred in the number of *S. symbiotica* cells in *C. tujafilina*, cell lysis does not appear to release putatively protective metabolites. Nevertheless, it is important to note that most enzymes involved in the metabolism of these compounds, which are encoded in the *S. symbiotica* SAp genome, are also retained in strain SCt, while they are completely absent in SCc (4, 23, 29). However, *pan*D, encoding aspartate 1-decarboxylase (EC 4.1.1.11), the protein involved in the biosynthesis of β-alanine from L-aspartate, has not been annotated in *S. symbiotica* SCt. Therefore, it is possible that the genome reduction undergone by this strain after its recent establishment as an obligate endosymbiont is already affecting its ability to protect the symbiotic consortium from heat stress. In any case, our results are only indirect evidence, and further studies using controlled experiments are needed in order to test the hypothesis.

In summary, we have performed a study on two populations belonging to two highly related conifer aphid species that present a *Buchnera/Serratia* consortium, but differ in the stage of their obligate relationship with the host. Our results indicate that *S. symbiotica* SCc, with a well-established co-obligate endosymbiotic status, has lost its ability to protect the aphid and *B. aphidicola* from heat stress. On the other hand *S. symbiotica* SCt, with an early stage of integration in its consortium with *B. aphidicola*, may still retain some protective role; however, the mechanisms responsible for this protection do not appear to involve cell lysis, as it was proposed in pea aphids. The difference between both consortia may explain the two aphid population dynamics through the annual seasons and may also make *C. cedri* more vulnerable to the increase expected in the frequency of extreme heat events in the Mediterranean area predicted by climate change models.

## Figures and Tables

**Table 1:** Primers designed for qPCR experiments.

Target	Specificity	Primer	Sequence (5′→3′)	Amplicon (bp)
*gro*EL	*B. aphidicola* BCc	groELF groELR	TACGTGCTATGGAAGCTCC GCAACAGAAGCAGCATATTGT	202
	*B. aphidicola BCt*			
*atp*D	*S. symbiotica* SCc	atpDSCc167F atpDSCc345R	GGTCTGATGAATGAACCAC GCTAAAGTAGGCTGATACC	196
	*S. symbiotica* SCt	atpDSCc267F atpD402R	GAACTGATCGTAACATCG GCTCATTCATCTGACCGTAA	155

**Table 2:** Experimental data from the *C. cedri* population. Temperature parameters (°C) were recorded the day before sampling.

Date	T_ave_	T_max_	T_min_	T_osc_	SD_T_	Aphid weight (mean ± SD) (mg)	*gro*EL (copies ng^−1^_T_DNA ± SD) (×10^6^)	*atp*D (copies ng^−1^_T_DNA ± SD) (×10^6^)
30/04/2012	15.30	22.80	11.40	11.40	3.47	0.59 ± 0.49	3.81 ± 3.21	0.26 ± 0.03
03/05/2012	16.15	21.20	10.70	10.50	3.67	0.88 ± 0.92	4.11 ± 0.87	0.70 ± 0.06
07/05/2012	18.89	24.70	13.90	10.80	3.11	1.11 ± 1.20	3.19 ± 0.66	0.53 ± 0.03
10/05/2012	22.27	27.20	17.20	10.00	3.28	0.44 ± 0.49	3.72 ± 0.53	0.36 ± 0.20
14/05/2012	24.71	32.00	18.30	13.70	4.23	0.93 ± 0.07	5.30 ± 1.70	1.03 ± 0.31
17/05/2012	19.13	23.50	13.70	9.80	2.92	0.98 ± 0.14	8.48 ± 0.88	0.97 ± 0.16
21/05/2012	20.07	25.30	15.30	10.00	2.93	0.72 ± 0.49	6.93 ± 1.45	0.58 ± 0.21
24/05/2012	22.31	28.90	14.90	14.00	4.43	0.89 ± 0.35	5.90 ± 2.35	0.50 ± 0.01
28/05/2012	22.41	26.80	19.30	7.50	2.54	1.02 ± 0.28	8.34 ± 0.90	0.94 ± 0.09
31/05/2012	23.15	29.20	17.90	11.30	3.39	0.94 ± 0.71	5.40 ± 1.22	0.63 ± 0.14
04/06/2012	24.41	29.30	21.10	8.20	2.29	0.68 ± 0.00	3.69 ± 0.31	0.44 ± 0.05
07/06/2012	25.88	33.40	18.90	14.50	4.63	1.80 ± 0.00	2.48 ± 0.10	0.29 ± 0.03
03/06/2013	20.42	25.10	14.80	10.30	3.50	1.16 ± 0.14	1.77 ± 0.09	0.38 ± 0.03
18/06/2013	24.08	29.20	19.20	10.00	2.97	1.22 ± 0.21	2.63 ± 0.41	0.50 ± 0.09
25/06/2013	21.97	24.60	19.10	5.50	1.59	1.08 ± 0.35	2.11 ± 0.46	0.50 ± 0.12
02/07/2013	23.45	28.80	17.00	11.80	3.67	0.86 ± 0.00	2.04 ± 0.48	0.36 ± 0.08

**Table 3:** Experimental data from the *C. tujafilina* population. Temperature parameters (°C) were recorded the day before sampling.

Date	T_ave_	T_max_	T_min_	T_osc_	SD_T_	Aphid weight (mean ± SD) (mg)	*gro*EL (copies ng^−1^_T_DNA ± SD) (×10^6^)	*atp*D (copies ng^−1^_T_DNA ± SD) (×10^6^)
03/05/2012	16.15	21.20	10.70	10.50	3.67	0.77 ± 1.41	2.20 ± 1.16	0.95 ± 0.28
07/05/2012	18.89	24.70	13.90	10.80	3.11	1.48 ± 1.63	1.45 ± 0.55	0.87 ± 0.33
10/05/2012	22.27	27.20	17.20	10.00	3.28	1.21 ± 0.07	1.31 ± 0.18	0.72 ± 0.18
14/05/2012	24.71	32.00	18.30	13.70	4.23	1.54 ± 0.64	3.62 ± 0.20	2.33 ± 0.78
17/05/2012	19.13	23.50	13.70	9.80	2.92	1.81 ± 0.49	2.37 ± 2.08	2.09 ± 2.87
21/05/2012	20.07	25.30	15.30	10.00	2.93	1.68 ± 0.14	3.17 ± 0.71	2.08 ± 0.57
24/05/2012	22.31	28.90	14.90	14.00	4.43	1.60 ± 0.14	3.73 ± 0.22	2.22 ± 0.14
28/05/2012	22.41	26.80	19.30	7.50	2.54	2.15 ± 0.49	4.19 ± 0.47	3.47 ± 0.52
31/05/2012	23.15	29.20	17.90	11.30	3.39	2.40 ± 0.14	4.49 ± 0.97	3.56 ± 0.75
04/06/2012	24.41	29.30	21.10	8.20	2.29	1.53 ± 0.64	3.25 ± 0.36	2.37 ± 0.35
07/06/2012	25.88	33.40	18.90	14.50	4.63	1.63 ± 0.00	3.64 ± 0.77	2.24 ± 0.57
23/07/2012	26.30	30.20	23.00	7.20	2.20	0.95 ± 0.07	0.95 ± 0.08	0.74 ± 0.09
15/10/2012	19.72	27.20	14.00	13.20	4.33	0.78 ± 0.07	1.16 ± 0.28	1.07 ± 0.16
18/10/2012	18.27	24.80	10.80	14.00	4.51	1.64 ± 0.35	1.71 ± 0.57	1.22 ± 0.31
22/10/2012	17.83	21.20	14.40	6.80	1.73	0.67 ± 0.07	1.21 ± 0.33	0.86 ± 0.12
25/10/2012	18.11	23.10	13.50	9.60	2.92	0.93 ± 0.00	1.63 ± 0.34	1.17 ± 0.38
29/10/2012	12.59	18.20	7.00	11.20	3.18	0.72 ± 0.14	1.08 ± 0.56	0.79 ± 0.32
05/11/2012	21.65	29.60	17.20	12.40	3.55	1.45 ± 0.07	2.08 ± 1.29	2.11 ± 1.51
08/11/2012	12.54	15.70	10.90	4.80	1.21	2.35 ± 0.07	1.75 ± 0.42	1.58 ± 0.26
12/11/2012	12.33	14.60	9.50	5.10	1.34	1.29 ± 0.07	1.44 ± 0.32	1.38 ± 0.25
15/11/2012	17.29	18.90	15.60	3.30	0.78	1.40 ± 0.14	0.78 ± 0.16	0.85 ± 0.13
19/11/2012	15.55	20.60	12.60	8.00	2.04	1.08 ± 0.00	0.84 ± 0.12	0.94 ± 0.15
22/11/2012	15.25	20.10	11.20	8.90	2.57	1.85 ± 0.14	2.32 ± 0.30	2.29 ± 1.20
26/11/2012	12.75	19.00	7.80	11.20	3.56	1.60 ± 0.28	1.43 ± 0.35	1.62 ± 0.78
29/11/2012	12.04	16.20	8.80	7.40	2.27	1.45 ± 0.07	1.66 ± 0.26	1.69 ± 0.10
03/12/2012	6.80	14.70	−0.30	15.00	4.92	1.19 ± 0.14	1.01 ± 0.10	1.13 ± 0.27
06/12/2012	11.52	17.80	6.10	11.70	3.32	1.01 ± 0.07	0.83 ± 0.13	0.79 ± 0.15
10/12/2012	7.24	15.80	1.70	14.10	4.74	1.26 ± 0.42	0.93 ± 0.14	0.66 ± 0.24
14/12/2012	10.45	17.90	3.90	14.00	4.96	0.81 ± 0.28	1.44 ± 0.37	1.40 ± 0.22
17/12/2012	16.68	19.50	14.10	5.40	1.34	1.28 ± 0.00	1.55 ± 0.25	2.22 ± 0.48
20/12/2012	13.26	19.70	9.20	10.50	3.42	1.27 ± 0.14	0.75 ± 0.15	1.05 ± 0.12
09/04/2013	15.73	22.30	9.60	12.70	3.80	0.86 ± 0.21	0.74 ± 0.24	0.71 ± 0.25
16/04/2013	17.31	24.00	10.70	13.30	4.71	0.59 ± 0.00	0.84 ± 0.30	0.71 ± 0.20
23/04/2013	14.37	19.20	8.60	10.60	3.43	0.80 ± 0.00	0.80 ± 0.14	0.82 ± 0.21
30/04/2013	11.35	15.70	8.30	7.40	2.06	0.98 ± 0.14	1.27 ± 0.16	1.22 ± 0.17
07/05/2013	18.86	26.30	10.60	15.70	4.84	1.33 ± 0.35	2.03 ± 1.54	2.45 ± 2.80
14/05/2013	18.46	23.80	13.40	10.40	3.13	0.82 ± 0.28	0.93 ± 0.19	0.91 ± 0.25
21/05/2013	14.92	19.20	10.70	8.50	2.23	0.58 ± 0.14	0.48 ± 0.13	0.56 ± 0.04
28/05/2013	16.43	20.10	12.80	7.30	1.94	0.53 ± 0.14	0.61 ± 0.12	0.43 ± 0.09
04/06/2013	19.90	24.80	13.80	11.00	3.52	0.48 ± 0.07	0.68 ± 0.07	0.43 ± 0.06
11/06/2013	23.62	31.10	16.70	14.40	3.99	0.53 ± 0.00	1.01 ± 0.11	0.66 ± 0.11
18/06/2013	24.08	29.20	19.20	10.00	2.97	1.19 ± 0.00	1.49 ± 0.37	1.06 ± 0.19
13/11/2013	17.48	24.10	11.40	12.70	3.87	0.48 ± 0.00	0.83 ± 0.04	0.29 ± 0.05

## References

[1] Altschul S.F., Madden T.L., Schäffer A.A., Zhang J., Zhang Z., Miller W., Lipman D.J. (1997). Gapped BLAST and PSI-BLAST: a new generation of protein database search programs. Nucleic Acids Res.

[2] Burke G., Fiehn O., Moran N. (2010). Effects of facultative symbionts and heat stress on the metabolome of pea aphids. ISME J.

[3] Burke G.R., Normark B.B., Favret C., Moran N.A. (2009). Evolution and diversity of facultative symbionts from the aphid subfamily *Lachninae*. Appl Environ Microbiol.

[4] Burke G.R., Moran N.A. (2011). Massive genomic decay in *Serratia symbiotica*, a recently evolved symbiont of aphids. Genome Biol Evol.

[5] Bustin S., Benes V., Garson J. (2009). The MIQE guidelines: minimum information for publication of quantitative real-time PCR experiments. Clin Chem.

[6] Chen D.Q., Montllor C.B., Purcell A.H. (2000). Fitness effects of two facultative endosymbiotic bacteria on the pea aphid, *Acyrthosiphon pisum*, and the blue alfalfa aphid, *A. kondoi*. Entomol Exp Appl.

[7] Degnan P.H., Ochman H., Moran N.A. (2011). Sequence conservation and functional constraint on intergenic spacers in reduced genomes of the obligate symbiont *Buchnera*. PLoS Genet.

[8] Douglas A.E. (1998). Nutritional interactions in insect-microbial symbioses: aphids and their symbiotic bacteria *Buchnera*. Annu Rev Entomol.

[9] Dunn A.M., Hogg J.C., Hatcher M.J. (2006). Transmission and burden and the impact of temperature on two species of vertically transmitted microsporidia. Int J Parasitol.

[10] Durak R., Borowiak-Sobkowiak B. (2013). Influence of temperature on the biological parameters of the anholocyclic species *Cinara tujafilina* (Hemiptera:Aphidoidea). Cent Eur J Biol.

[11] Durak R. (2014). Life cycle, seasonal and interannual polymorphism in a monoecious aphid *Cinara mordvilkoi* (Hemiptera:Aphidoidea: Lachnidae). Eur J Entomol Entomol.

[12] Engelstädter J., Hurst G.D.D. (2009). The ecology and evolution of microbes that manipulate host reproduction. Annu Rev Ecol Evol Syst.

[13] Feldhaar H. (2011). Bacterial symbionts as mediators of ecologically important traits of insect hosts. Ecol Entomol.

[14] Ferrari J., Scarborough C.L., Godfray H.C.J. (2007). Genetic variation in the effect of a facultative symbiont on host-plant use by pea aphids. Oecologia.

[15] Gosalbes M.J., Lamelas A., Moya A., Latorre A. (2008). The striking case of tryptophan provision in the cedar aphid *Cinara cedri*. J Bacteriol.

[16] Hansen A.K., Moran N.A. (2011). Aphid genome expression reveals host-symbiont cooperation in the production of amino acids. Proc Natl Acad Sci USA.

[17] Harmon J.P., Moran N.A., Ives A.R. (2009). Species response to environmental change: impacts of food web interactions and evolution. Science.

[18] Henry L.M., Peccoud J., Simon J.C., Hadfield J.D., Maiden M.J.C., Ferrari J., Godfray H.C.J. (2013). Horizontally transmitted symbionts and host colonization of ecological niches. Curr Biol.

[19] Köhler M., Schwartz W. (1962). Untersuchungen über die symbiose von tieren mit pilzen und bakterien. IX. Über die beziehungen zwischen symbionten und wirtsorganismus bei *Pseudococcus citri, Ps. maritimus* und *Orthezia insignis*. Z Allg Mikrobiol.

[20] Komaki K., Ishikawa H. (1999). Intracellular bacterial symbionts of aphids possess many genomic copies per bacterium. J Mol Evol.

[21] Komaki K., Ishikawa H. (2000). Genomic copy number of intracellular bacterial symbionts of aphids varies in response to developmental stage and morph of their host. Insect Biochem Mol Biol.

[22] Lamelas A., Pérez-Brocal V., Gómez-Valero L., Gosalbes M.J., Moya A., Latorre A. (2008). Evolution of the secondary symbiont “*Candidatus* Serratia symbiotica” in aphid species of the subfamily *Lachninae*. Appl Environ Microbiol.

[23] Lamelas A., Gosalbes M.J., Manzano-Marín A., Peretó J., Moya A., Latorre A. (2011). *Serratia symbiotica* from the aphid *Cinara cedri*: a missing link from facultative to obligate insect endosymbiont. PLoS Genet.

[24] Lamelas A., Gosalbes M.J., Moya A., Latorre A. (2011). New clues about the evolutionary history of metabolic losses in bacterial endosymbionts, provided by the genome of *Buchnera aphidicola* from the aphid *Cinara tujafilina*. Appl Environ Microbiol.

[25] Larkin M.A., Blackshields G., Brown N.P. (2007). Clustal W and Clustal X version 2.0. Bioinformatics.

[26] Latorre A., Moya A., Ayala F.J. (1986). Evolution of mitochondrial DNA in *Drosophila subobscura*. Proc Natl Acad Sci USA.

[27] Lee C., Kim J., Shin S.G., Hwang S. (2006). Absolute and relative qPCR quantification of plasmid copy number in *Escherichia coli*. J Biotechnol.

[28] MacDonald S.J., Thomas G.H., Douglas A.E. (2011). Genetic and metabolic determinants of nutritional phenotype in an insect-bacterial symbiosis. Mol Ecol.

[29] Manzano-Marín A., Latorre A. (2014). Settling down: the genome of *Serratia symbiotica* from the aphid *Cinara tujafilina* zooms in on the process of accommodation to a cooperative intracellular life. Genome Biol Evol.

[30] Montllor C.B., Maxmen A., Purcell A.H. (2002). Facultative bacterial endosymbionts benefit pea aphids *Acyrthosiphon pisum* under heat stress. Ecol Entomol.

[31] Moran N.A., Munson M.A., Baumann P., Ishikawa H. (1993). A molecular clock in endosymbiotic bacteria is calibrated using the insect hosts. Proc R Soc B Biol Sci.

[32] Moran N.A., Russell J.A., Koga R., Fukatsu T. (2005). Evolutionary relationships of three new species of *Enterobacteriaceae* living as symbionts of aphids and other insects.

[33] Mouton L., Henri H., Charif D., Boulétreau M., Vavre F. (2007). Interaction between host genotype and environmental conditions affects bacterial density in *Wolbachia* symbiosis. Biol Lett.

[34] Moya A., Peretó J., Gil R., Latorre A. (2008). Learning how to live together: genomic insights into prokaryote-animal symbioses. Nat Rev Genet.

[35] Nieto-Nafría J.M., Mier-Durante M.P., Binazzi A., Pérez-Hidalgo N., Ramos M.A. (2003). Hemiptera, Aphididae II. Fauna Iberica.

[36] Ohtaka C., Ishikawa H. (1991). Effects of heat-treatment on the symbiotic system of an aphid mycetocyte. Symbiosis.

[37] Oliver K.M., Russell J.A., Moran N.A., Hunter M.S. (2003). Facultative bacterial symbionts in aphids confer resistance to parasitic wasps. Proc Natl Acad Sci USA.

[38] Oliver K.M., Moran N.A., Hunter M.S. (2006). Costs and benefits of a superinfection of facultative symbionts in aphids. Proc Biol Sci.

[39] Oliver K.M., Degnan P.H., Burke G.R., Moran N.A. (2010). Facultative symbionts in aphids and the horizontal transfer of ecologically important traits. Annu Rev Entomol.

[40] Oliver K.M., Smith A.H., Russell J.A. (2014). Defensive symbiosis in the real world—dvancing ecological studies of heritable, protective bacteria in aphids and beyond. Funct Ecol.

[41] Pérez-Brocal V., Gil R., Ramos S., Lamelas A., Postigo M., Michelena J.M., Silva J.F., Moya A., Latorre A. (2006). A small microbial genome: the end of a long symbiotic relationship?. Science.

[42] Poliakov A., Russell C.W., Ponnala L., Hoops H.J., Sun Q., Douglas A.E., van Wijk K.J. (2011). Large-scale label-free quantitative proteomics of the pea aphid-*Buchnera* symbiosis. Mol Cell Proteomics.

[43] Russell J.A., Latorre A., Sabater-Muñoz B., Moya A., Moran N.A. (2003). Side-stepping secondary symbionts: widespread horizontal transfer across and beyond the *Aphidoidea*. Mol Ecol.

[44] Russell J.A., Moran N.A. (2006). Costs and benefits of symbiont infection in aphids: variation among symbionts and across temperatures. Proc Biol Sci.

[45] Scarborough C.L., Ferrari J., Godfray H.C.J. (2005). Aphid protected from pathogen by endosymbiont. Science.

[46] Shigenobu S., Watanabe H., Hattori M., Sakaki Y., Ishikawa H. (2000). Genome sequence of the endocellular bacterial symbiont of aphids *Buchnera* sp. APS Nature.

[47] Tamas I., Klasson L., Canbäck B., Näslund A.K., Eriksson A.-S., Wernegreen J.J., Sandström J.P., Moran N.A., Andersson S.G.E. (2002). 50 million years of genomic stasis in endosymbiotic bacteria. Science.

[48] Thomas M.B., Blanford S. (2003). Thermal biology in insect-parasite interactions. Trends Ecol Evol.

[49] Tsuchida T., Koga R., Fukatsu T. (2004). Host plant specialization governed by facultative symbiont. Science.

[50] van Ham R.C.H.J., Kamerbeek J., Palacios C. (2003). Reductive genome evolution in *Buchnera aphidicola*. Proc Natl Acad Sci USA.

[51] Wernegreen J.J. (2012). Mutualism meltdown in insects: bacteria constrain thermal adaptation. Curr Opin Microbiol.

[52] Yakovchuk P., Protozanova E., Frank-Kamenetskii M.D. (2006). Base-stacking and base-pairing contributions into thermal stability of the DNA double helix. Nucleic Acids Res.

[53] Zytynska S.E., Weisser W.W. (2016). The natural occurrence of secondary bacterial symbionts in aphids. Ecol Entomol.

